# Case Report: Successful Chimeric Antigen Receptor T Cell Therapy in Haploidentical-Allogeneic Stem Cell Transplant Patients With Post-Transplant Lymphoproliferative Disorder

**DOI:** 10.3389/fonc.2021.709370

**Published:** 2021-07-22

**Authors:** Nan Yan, Na Wang, Peiling Zhang, Gaoxiang Wang, Xia Mao, Dan Peng, Dong Kuang, Liting Chen, Li Zhu, Jianfeng Zhou, Yicheng Zhang, Yang Cao

**Affiliations:** ^1^ Department of Hematology, Tongji Hospital, Tongji Medical College, Huazhong University of Science and Technology, Wuhan, China; ^2^ Immunotherapy Research Center for Hematologic Diseases of Hubei Province, Wuhan, China; ^3^ Department of Nuclear Medicine, Tongji Hospital, Tongji Medical College, Huazhong University of Science and Technology, Wuhan, China; ^4^ Institute of Pathology, Tongji Hospital, Tongji Medical College, Huazhong University of Science and Technology, Wuhan, China

**Keywords:** CAR-T, HSCT, PTLD, EBV, ALL

## Abstract

**Background:**

Epstein–Barr virus-associated post-transplant lymphoproliferative disorder (EBV-PTLD) is a potentially fatal complication after allogeneic hematopoietic stem cell transplantation (allo-HSCT). Rituximab has been proven to dramatically improve the prognosis of patients with EBV reactivation and PTLD. However, reports on the curative management of refractory PTLD are scarce.

**Case Presentation:**

In this report, we describe the successful management of two patients with EBV-PTLD with chimeric antigen receptor T-cell (CAR-T) therapy.

**Conclusion:**

The present results demonstrated that patients with EBV-PTLD may benefit from CAR-T therapy and that the toxicity is manageable. Further studies are needed to verify these findings.

## Introduction

Post-transplant lymphoproliferative disorder (PTLD) is characterized by lymphoid or plasmacytic proliferation that develops as a consequence of immunosuppression in a recipient after allo-HSCT or solid organ transplantation, and PTLD is regarded as one of the most serious post-transplantation complications due to its high mortality (1, 2). PTLD in the HSCT setting is almost exclusively related to Epstein–Barr virus (EBV) infection (3). The most common symptoms and signs of EBV-PTLD are prolonged fever and lymphadenopathy, which may rapidly progress to multiorgan failure and even death.

An effective strategy against EBV reactivation in the HSCT setting is based on weekly quantitative PCR scanning and timely introduced pre-emptive therapy of a reduction in immunosuppression (RIS) or rituximab (4). Most conventional treatment options can be effective for 60–80% of PTLD. For patients with a poor response, treatment options are limited and call for more effective treatments. Adoptive cell therapy with T cells genetically engineered to express chimeric antigen receptor (CAR) targeting CD19 represents a promising approach in treating relapsed/refractory (r/r) B-cell malignancies (5). Clinical trials of CD19-specific CAR-T cells have shown complete remission rates of 70 to 90% among patients with r/r B-cell acute lymphoblastic leukemia (B-ALL) and 50% among patients with r/r B-cell non-Hodgkin lymphoma (B-NHL) (6–10). However, antigen-escape relapse represents one of the most frequent causes of treatment failure (11–13). To reduce the possibility of relapse due to target antigen loss and/or mutation, dual antigen-targeted CAR-T cells are being developed, and clinical trials have elicited excellent responses (14, 15). Based on the results of our previous clinical trial with CD19/22 cocktail therapy in r/r malignancies, here, we present the first report of the use of sequential CD19 and CD22 CAR-T cell therapies in haploidentical HSCT in two patients with early-onset EBV-PTLD.

## Case Report

### Case 1

**Figure 1:**
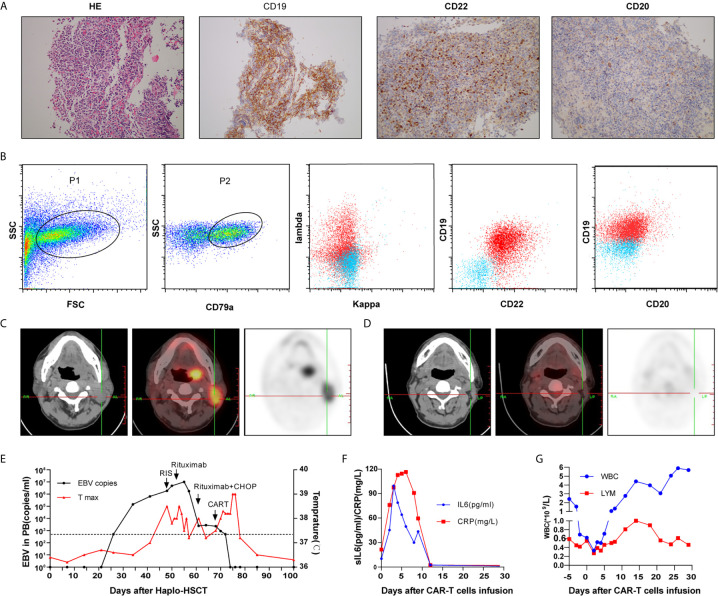
Cervical lymph node biopsy **(A)** and immunophenotypic analysis **(B)** of case 1 by flow cytometry revealed a CD20-negative EBV-PTLD; Computed tomography and positron emission tomography images before **(C)** and after treatment **(D)**; Timeline of EBV PCR and clinical data **(E)**; CRP, sIL6 **(F)** and peripheral hemogram **(G)** after CAR-T infusion.

A 30-year-old male was referred to our hospital because of his refractory B-ALL. He underwent three cycles of induction chemotherapy (VTCLP: Vincristine; Tepirubicin; Cyclophosphamide; PEG-Asparaginase; Prednisone/VICP: Vincristine; Idarubicin; Cyclophosphamide; Prednisone/hyper CVAD B: Methotrexate; Cytarabine) but failed to achieve remission. On admission, he had severe pneumonitis. Bone marrow and peripheral blood smears identified the proliferation of lymphoblastic cells (87% of bone marrow nucleated cells), and karyotyping revealed an abnormal complex karyotype. NGS was performed and TP53 mutation was identified. After controlling his infection, he was recruited for a CAR-T clinical trial (Chictr.org N, ChiCTR-OPN-16008526) under informed consent in October 2017. The preparation of autologous CAR-T cells is described in detail in the **Supplementary Material**. The patient received cyclophosphamide 300 mg/m^2^ and fludarabine 25 mg/m^2^ conditioning regimen on days −4 to −2 prior to CAR-T infusion. Anti-CD22 CAR-T cells (2 × 10^6^/kg) and anti-CD19 CAR-T cells (2 × 10^6^/kg) were infused on two consecutive days. He developed Grade 3 cytokine release syndrome (CRS). The minimal residual leukemia on day +28 after CAR-T infusion examined by FCM was negative. On April 13, 2018, the patient underwent allogeneic transplantation with granulocyte colony-stimulating factor mobilized bone marrow cells plus peripheral blood hematopoietic stem cells from his cousin, who was HLA-five loci mismatched. He received a TBI/VP/CY/ATG conditioning regimen consisting of total body irradiation (day −10), etoposide (15 mg/kg/day on days −8 and −7), cyclophosphamide (1.8 g/m^2^/day on days −6 and −5), and ATG (thymoglobulin, 2.0 mg/kg/day on days −4 to −1). Prophylaxis for GVHD consisted of cyclosporine and MMF (from day −1) and short-term methotrexate on days 1, 3, 6, and 11. The engraftment of neutrophils was achieved on day +11. Chimeric analysis showed the complete donor type on day 28, and no GVHD occurred. Quantitative EBV PCR monitoring was performed weekly. On day 34, measurement of the EBV-DNA level showed an increase to 143,000 copies/ml (from <500 copies/ml on day 27). A reduction in immunosuppression (the cessation of MMF, cyclophosphamide dose reduction) in combination with rituximab (375 mg/m^2^) weekly was initiated for the patient. However, his EBV-DNA copy numbers in PBMCs continued to increase, and subsequently, he developed fever and enlarged lymph nodes in the neck on day 50. A biopsy of the mass showed that the normal structure was destroyed, with diffuse invasion of cells showing atypical nuclear bodies, and immunostaining positivity for CD19, CD22, PAX5, MUM-1 and EBER; in addition approximately 80% of the tumor cells showed positive Ki-67 staining. However, CD20 was almost negative (**Figures 1A, B**). PET-CT detected hypermetabolic lesions and the involvement of the 86 cervical lymph nodes (**Figure 1C**). EBV-PTLD (CD20 negative) was diagnosed on the basis of the results. Given to the poor prognosis and the prominent presence of EBV, immunosuppression was completely discontinued, and CHOP (cyclophosphamide, doxorubicin, vincristine and prednisone) was given. However, his EBV viral load was still more than 10^3^ copies/ml. Considering the insufficient number of T lymphocytes after CHOP therapy, donor T cells were collected for CAR-T preparation. Then, he received a total dose of 2 × 10^6^/kg anti-CD22 and 2 × 10^6^/kg anti-CD19 donor-derived CAR-T infusion from days 68 to 69. The timelines of EBV PCR and related treatments are depicted in **Figure 1E**. On day 4 post-CAR-T infusion, the patient had grade 1 CRS presenting as fever and slightly elevated CRP/sIL6 (**Figure 1F**), resolved fully by symptomatic and supportive treatment. He developed acute GVHD (grade II) on day 78, and the administration of prednisolone at 1.0 mg/kg was initiated. Although grade 4 neutropenia and thrombocytopenia were prolonged for approximately 2 weeks (**Figure 1G**), the patient’s cervical lymphadenopathy improved and responded well to CAR-T therapy. After recovery from myelosuppression, PET-CT showed a resolution of the cervical lymphadenopathy (**Figures 1D**), and a rapid reduction in the blood EBV load was observed (400 copies/ml). EBV-DNA could not be detected in the plasma during the follow-up.

Unfortunately, the patient continued to have multidrug-resistant pneumonia and elected to pursue hospice care, where he subsequently passed away on day 300 post HSCT.

### Case 2

**Figure 2:**
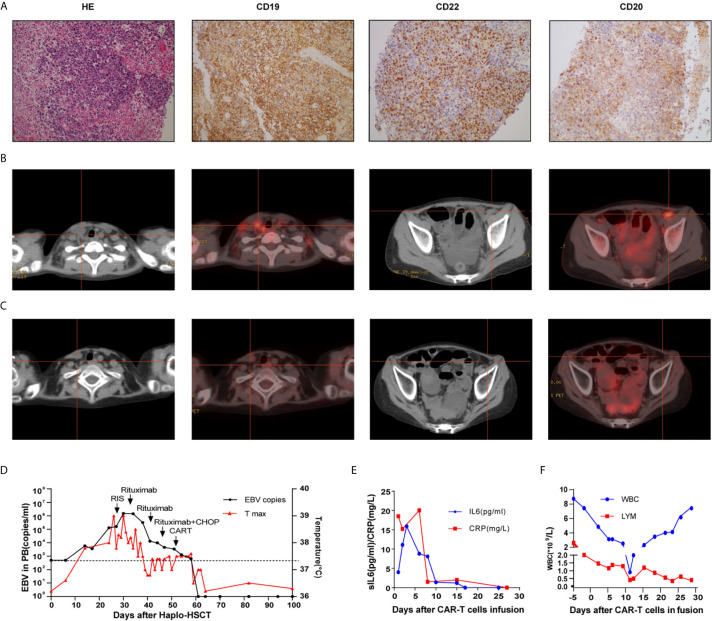
Cervical lymph node biopsy of case 2 **(A)**; Computed tomography and positron emission tomography images before **(B)** and after treatment **(C)**;Timeline of EBV PCR and clinical data **(D)**; CRP, sIL6 **(E)** and peripheral hemogram **(F)** after CAR-T infusion.

A 10-year-old girl was diagnosed with B-ALL in 2014. She had abnormal complex karyotypes, and no fusion genes were detected. Although she received standard chemotherapy according to the CCCG-ALL 2008 protocol (16), her ALL relapsed in 2017. Reinduction chemotherapy failed to induce a CR. She was also recruited for a CAR-T clinical trial under informed consent in December 2017. After recruitment, autologous peripheral-blood mononuclear cells were collected by means of apheresis before the administration of lymphocyte-depleting chemotherapy (cyclophosphamide 300 mg/m^2^ and fludarabine 25 mg/m^2^ conditioning regimen on days −4 to −2). The related preparation of CAR-T cells is shown in the **Supplemental Material**. She received an infusion of anti-CD22 CAR-T cells (2 × 10^6^/kg) and anti-CD19 CAR-T cells (2 × 10^6^/kg) on two consecutive days. After CAR-T infusion, she developed intermittent fever, but no severe CRS was observed. She exhibited a recovery of neutrophil count on day 16 after the infusion of CAR-T cells; her bone marrow smear showed complete remission. Because no HLA fully matched donors were found from either relatives or the Chinese bone marrow donor program, she received an allogeneic peripheral blood stem cell transplantation (PBSCT) from her 36-year-old father, who was HLA-four loci mismatched, in May 2018. She received a myeloablative conditioning regimen of 2 g/m^2^ cytarabine (days −9 and −8), 3.2 mg/kg busulfan (days −7 to −5), 1.8 g/m^2^ cyclophosphamide (days −4 and −3) and 2.0 mg/kg ATG (thymoglobulin, days −4 to −1). Prophylaxis for GVHD consisted of cyclosporine and MMF (from day −1) and short-term methotrexate (days +1,3, 6,11). There were no grade ≥3 adverse events other than hematological toxicity and febrile neutropenia until neutrophil engraftment. The engraftment of neutrophils was achieved on day 13, and the EBV-DNA copy number in PBMCs was 6,000 copies/ml. The grade II acute GVHD of the skin and fever that developed on day 24 were controlled by steroids. At this time point, her EBV-DNA copy number increased to 10^5^ copies/ml. Chimerism analysis showed the complete donor type on day 29. Persistent fever and systemic lymphadenopathy were observed after day 32, and increased lactate dehydrogenase (LDH 594 U/l) level and a high blood EBV load (10^6^ copies/ml) were observed. PET-CT detected hypermetabolic lesions and the involvement of several nodal groups. Lymph node biopsy histologically verified CD19, CD22 and CD20 positive B cell lymphoma with mixed cellularity, consistent with PTLD (**Figures 2A, B**). Furthermore, tumor-associated EBV was detected by EBER *in situ* hybridization.

Treatment with cyclophosphamide was slowly tapered without exacerbating GVHD. The patient was treated with rituximab weekly (375 mg/m^2^) from day 33; however, her fever and cervical lymphadenopathy continued. After two administrations of rituximab, half-dose treatment with R-CHOP (rituximab, cyclophosphamide, vincristine, doxorubicin, and prednisolone) was initiated on day 46. Despite these treatments, her EBV viral load was still 10^4^ copies/ml. Considering possible T cell dysfunction owing to the immunosuppressive agent used to control GVHD and insufficient T lymphocytes post-chemotherapy, donor lymphocytes were collected for CAR-T preparation, and the details are shown in the **Supplemental Material**. She received an anti-CD19 and anti-CD22 donor-derived CAR-T infusion at a dose of 2 × 10^6^/kg on days 53 and 54, respectively. After CAR-T infusion, she developed mild fever, grade 4 neutropenia and thrombocytopenia. She had an excellent response to CAR-T therapy, with rapidly reduced lymphadenopathy and a decline in EBV viral load to undetectable levels after recovery from myelosuppression, which lasted 10 days. The follow-up PET-CT scan showed complete metabolic remission (**Figure 2C**). The timelines of EBV PCR and related treatments were shown in **Figure 2D**. CRP, sIL6 and peripheral hemogram after CAR-T infusion were summarized in **Figures 2E, F**. EBV-DNA could not be detected in the plasma up to 24 weeks of follow-up.

## Discussion

The incidence of PTLD has increased in the last few decades; however, treatments for PTLD are limited, and the survival of PTLD patients remains inferior. In a study by the European Group for Blood and Marrow Transplantation, the overall incidence of PTLD after HSCT was found to be 3.22%, ranging from 1.16 to 11.24% in different HSCT settings; the cumulative mortality due to PTLD was 30.8% (17). To our knowledge, this is the first report of successful chimeric antigen receptor T cell therapy in haploidentical-allogeneic stem cell transplant patients with PTLD. Both patients achieved complete remission after receiving donor-derived CAR-T cell infusions, which was different from the cases previously reported that refractory EBV-negative PTLD in three solid organ recipients did not respond to CAR-T therapy, possibly due to different pathogeneses of EBV-positive and EBV-negative cases (18).

EBV-PTLD usually develops within the first 6 months after HSCT, which is before the reconstitution of EBV-specific cytotoxic T-cell immunity. Multiple risk factors that increase the likelihood of developing EBV-PTLD post HSCT have been elucidated, including T cell depletion, the use of anti-thymocyte globulin (ATG), the use of reduced intensity conditioning, donors other than HLA-matched related donors, pre-transplantation splenectomy, patient age ≥50 years, and acute and chronic graft-versus-host disease (GVHD) (19). Previous studies showed that the risk of developing PTLD increases with the accumulating risk factors (19). As haplo-HSCT recipients, our patients may have had more risk factors for PTLD, such as HLA disparity and the use of ATG and GVHD.

Generally, standard initial treatment of patients with PTLD includes a reduction in immunosuppression or the administration of rituximab. After the failure of the abovementioned treatment modalities, systemic chemotherapy with or without rituximab was recommended (20). In this study, our patients were characterized by early onset, aggressive clinical courses, and poor responses to conventional treatment. Despite a RIS and treatment with systemic rituximab and chemotherapy, both patients failed to achieve a complete response. Notably, one patient was CD20-negative and refractory to rituximab, and the other patient developed grade II acute GVHD at PTLD diagnosis, which was associated with worse OS and PTLD related mortality according to the survival prognostic model proposed by Styczynski et al. (17). To date, no standard therapy has been accepted for rituximab-resistant EBV-PTLD. As one of the second-line therapy options, cellular therapy, including donor lymphocyte infusion (DLI) and cytotoxic lymphocytes (CTLs), has shown promising results in treating EBV-PTLD, but DLI appeared ineffective as a salvage treatment for rituximab failure according to observations from two different centers (21, 22), and EBV-specific T cells are often not available since the production of EBV-CTLs could be costly and time-consuming. In addition, with T lymphocytes being involved in the pathogenesis of GVHD, the use of EBV-CTLs or DLI may increase the risk of the occurrence of GVHD. As one breakthrough technology in treating refractory hematological malignancies, CD19-directed CAR-T cell immunotherapies for the treatment of aggressive B-cell malignancies have been shown to elicit high overall response rates and CR rates in pivotal trials with mild and reversible side effects (23). However, a large proportion of CR patients relapsed within 1 year, and a loss of or mutation in CD19 is frequently observed and considered a major mechanism of relapse (6, 7, 24). CD19/CD22-bispecific CAR-T cells and sequential infusion of CD19/CD22 CAR-T cell have been used to prevent leukemia antigen loss. Clinical trials on bispecific CAR-T cells have demonstrated preliminary efficacy, but further structural optimization and dosage regimen exploration are ongoing (25–27). According to a study conducted by our center, sequential infusion of dual-target CAR-T cells can effectively reduce the recurrence caused by antigen loss in r/r B-cell malignancies, with a median PFS of 13.6 months (14). A phase I trial of sequential CD19 and CD22 CAR-T cell therapies in 20 refractory or relapsed B-ALL pediatric patients showed that 17/20 patients remained in remission at 4 to 20 months after the first infusion (15). Sequential infusion of CD19 and CD22 CAR T-cells is effective and safe in treating r/r B-cell malignancies and can improve the durability of remission in the long term. With dual expression of CD19 and CD22 on the surface of tumor cells, both patients received sequential infusion of CD19/CD22 CAR-T cell. Lymphadenopathy did not recur in our patients, and the blood EBV load remained negative during the follow up.

Moreover, preliminary results from several clinical trials show that donor CAR-T cells exert potent GVL activity in the absence of damaging GVHD activity (28–30). Donor-derived CAR-T cells and recipient-derived CAR-T cells seem to have similar efficacy and safety, probably owing to the same original allogenic donor immune system, although larger clinical studies are needed (31, 32). Considering insufficient T lymphocytes post-chemotherapy and possible T cell dysfunction owing to the immunosuppressive agent used to control GVHD, donor T cells were collected for CAR-T cell preparation. After donor-derived CAR-T infusion, there was no severe CRS in either patient. One patient later developed only grade II acute GVHD that could be controlled by prednisolone, demonstrating that donor-derived CAR-T cells might be considered a feasible and relatively safe option for EBV-PTLD post HSCT.

In summary, our results showed that CAR-T therapy could be an effective and tolerable treatment for PTLD with a significant effect and favorable toxicity profile, especially when initial therapy fails. Donor-derived CAR-T cells can be chosen for PTLD patients with insufficient T lymphocytes or T cell dysfunction. A larger cohort of patients is needed to further establish a proof of concept.

## Data Availability Statement

The original contributions presented in the study are included in the article/**Supplementary Material**. Further inquiries can be directed to the corresponding author.

## Ethics Statement

The studies involving human participants were reviewed and approved by the Ethical Review Committee of Tongji Hospital, Tongji Medical College, Huazhong University of Science and Technology. Written informed consent to participate in this study was provided by the participants’ legal guardian/next of kin.

## Author Contributions

YC designed and supervised the clinical study. NY and NW collected the clinical data. NY, NW, and YC analyzed the data and wrote the manuscript. YC, NW, PZ, GW, JZ, and YZ enrolled and took care of the patients. XM, LZ, LC, DP, and DK performed the laboratory tests and monitored the responses of the patients. All authors contributed to the article and approved the submitted version.

## Funding

This work was supported by the National Natural Science Foundation of China (81600120, to NW; 81570197, to YC).

## Conflict of Interest

The authors declare that the research was conducted in the absence of any commercial or financial relationships that could be construed as a potential conflict of interest.
