# Supplementary material for: Case Report: Successful Chimeric Antigen Receptor T Cell Therapy in Haploidentical-Allogeneic Stem Cell Transplant Patients With Post-Transplant Lymphoproliferative Disorder
Source: Front Oncol. 2021 Jul 22;11:709370. doi: 10.3389/fonc.2021.709370 (PMC8339712; doi:10.3389/fonc.2021.709370)
Supplement: Supplementary file 1 [file DataSheet_1.docx]

Supplement to ***Nan Yan, et al. Successful chimeric antigen receptor T cells therapy in haploidentical-allogeneic stem cell transplant patients with post‐transplant lymphoproliferative disorder***

**Supplementary Data**

**Methods**

**Supplementary Figure 1**

**Supplementary Figure 2**

**Supplementary Figure 3**

**Supplementary References**

**Methods**

***CAR-T cell production***

CAR-T cells was prepared as previously reported^1,2^. Briefly, PBMC were isolated from peripheral blood from the patients or the donors by density gradient centrifugation, and T cells were separated from PBMC subsequently by CD3 microbeads (Miltenyi Biotec) and then stimulated with Dynabeads™ Human T-Activator CD3/CD28 (Invitrogen) in CTS™ OpTmizer™ medium (Gibco) containing 2 mM L-glutamine (Gibco) and 200IU/ml rhIL-2. Within 24 hours, the T cells were transduced separately with different lentivirus encoding anti-CD19 or anti-CD22 CAR at a multiplicity of infection (MOI) ranging from 1 to 5 and then amplification in the fresh culture medium. The transduction efficiency and immunophenotype of CAR-T cells were determined by flow cytometer and tumor cytotoxic effects of CAR-T cells was conducted by calcein release assay and cytokines release assay prior to infusion.

***Cell staining and The immunophenotype of CAR-T cells***

Cells was stained with Alexa Fluor 488 labeled rabbit anti-mouse-F(ab)_2_ antibody, then washed with PBS twice and then stained with anti-human CD3, CD4, CD8, CD45RO and CCR7(BD Biosciences). The cells were washed and resuspended in PBS and then determined by flow cytometry. The data was analyzed by Flowjo.

***Calcein release assay***

Tumor cytotoxic effects of CAR-T cells was conducted by calcein release assay as reported previously^3,4^. Positive target cells (Raji) were labeled with calcein (Aladdin) and then co-cultured with effector cells (anti-CD19 or anti-CD 22 CAR-T cells) and control T cells in 96-well plates at different ratios. After 4h of incubation, the cultures were centrifuged and the supernatants were transferred to another 96-well plate. The fluorescence value of each well (F) was determined with a microplate reader. The tumor-killing efficiency was calculated according to the formula: lysis% = (F_experimental well_ - F_spontaneous release_) / (F_maximum release_ - F_spontaneous release_) × 100%. (Wells with co-cultured target cells and lysis solution were used as maximum release wells, and wells with co-cultured target cells and PBS were used as spontaneous release wells.)

***Cytokine ralease assay***

Anti- CD19 or anti-CD 22 CAR-T cells were co-cultured with target cells(Raji) at the ratio of 10:1 for 24h. The culture medium was with no IL-2 added. Then cell culture supernatants were collected and the procedures were conducted according to the manufacturer’s instructions for the human IL-2, IL-6, TNF-α, IFN-γ ELISA kit(NEOBIOSCIENCE).


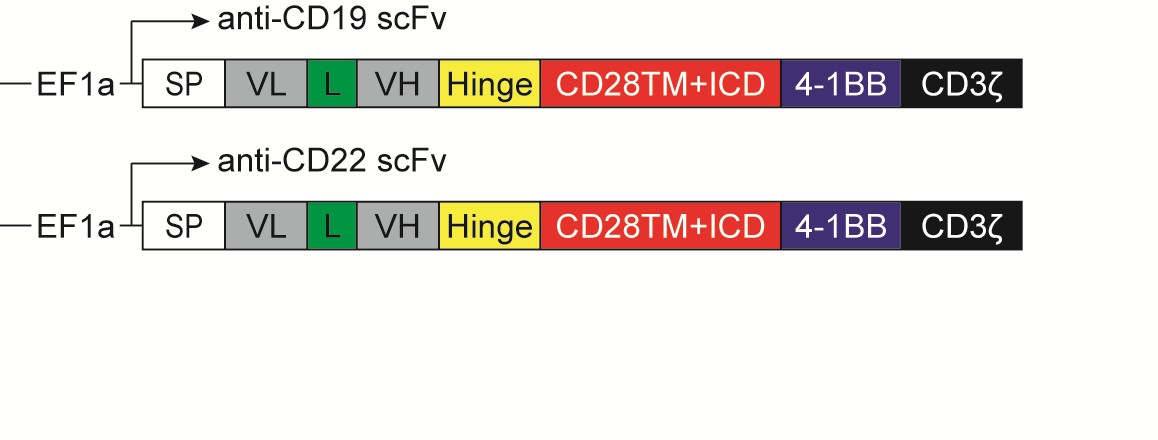


**Figure S1 . Schematic diagrams of anti-CD19 CAR and anti-CD22 CAR.** The third-generation CAR was utilized in this trial . A single chain variable fragment (scFv) was derived from a murine monoclonal antibody against human CD19 or CD22, two costimulatory domains was CD28 ICD and 4-1BB, and activation domain was CD3ζ chain. SP, signal peptide; VL, variable L chain; L, linker; VH, variable H chain.


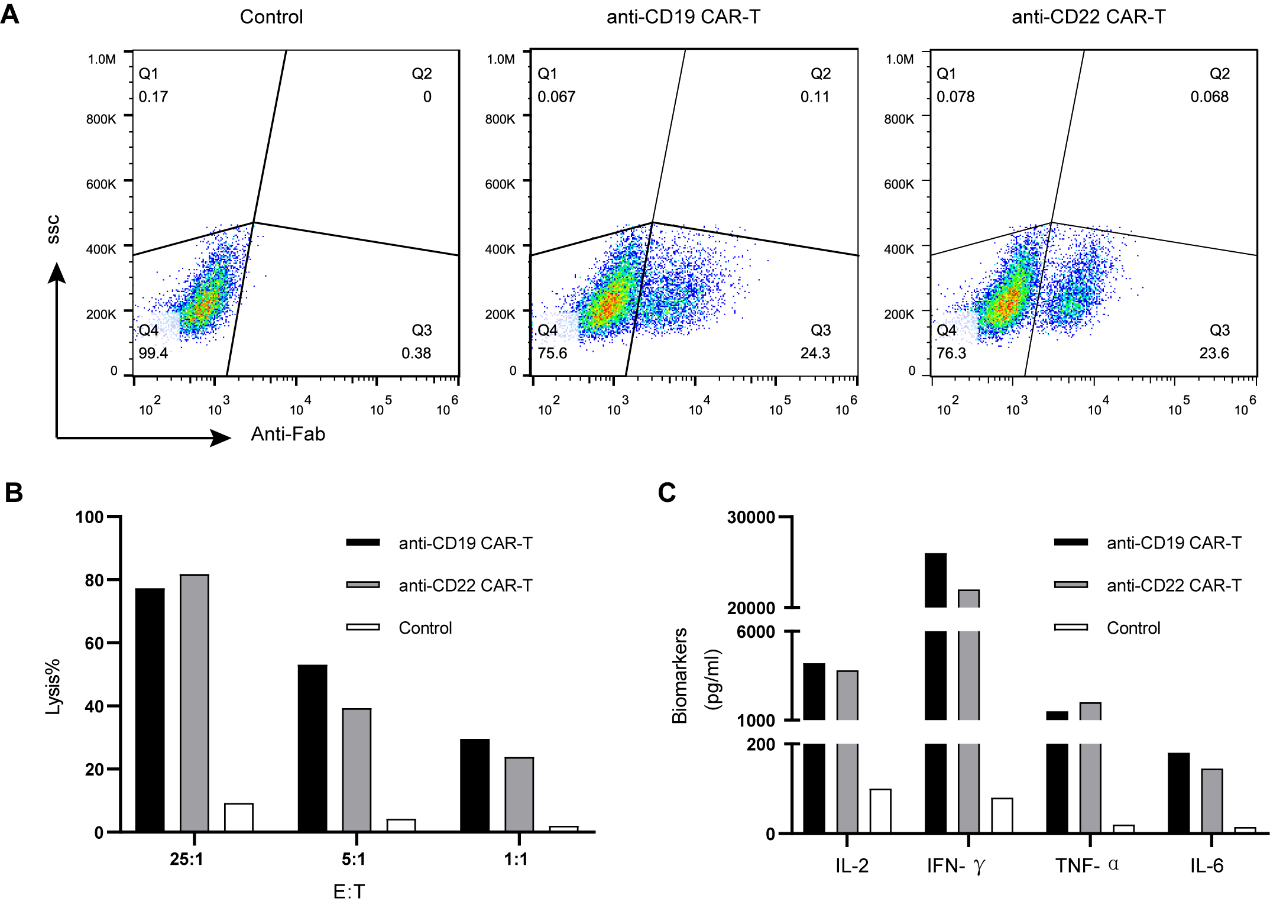


**Figure S2 . Transduction and antileukemic effects of anti-CD19 CAR-T and anti-CD22 CAR-T cells**. (A) Transfection efficiency of CAR-T cells was determined by flow cytometry by staining with Alexa Fluor 488 labeled rabbit anti-mouse-F(ab)_2_ antibody at day10 post transduction, 24.3% of anti-CD19 CAR-T cells and 23.8% of anti-CD22 CAR-T cells were transduced respectively; (B) Specific killing was evaluated at 4 hours after co-culture of effector T cells (CAR-T cells) with target cells (Raji cells) at different effector to target ratio (E:T); (C) Cytokines production of IL-2, IFN-γ, TNF-α and IL-6 was assessed at 24 hours after co-culture of effector T cells (CAR-T cells) with target cells (Raji cells) at an E:T ratio of 10:1;


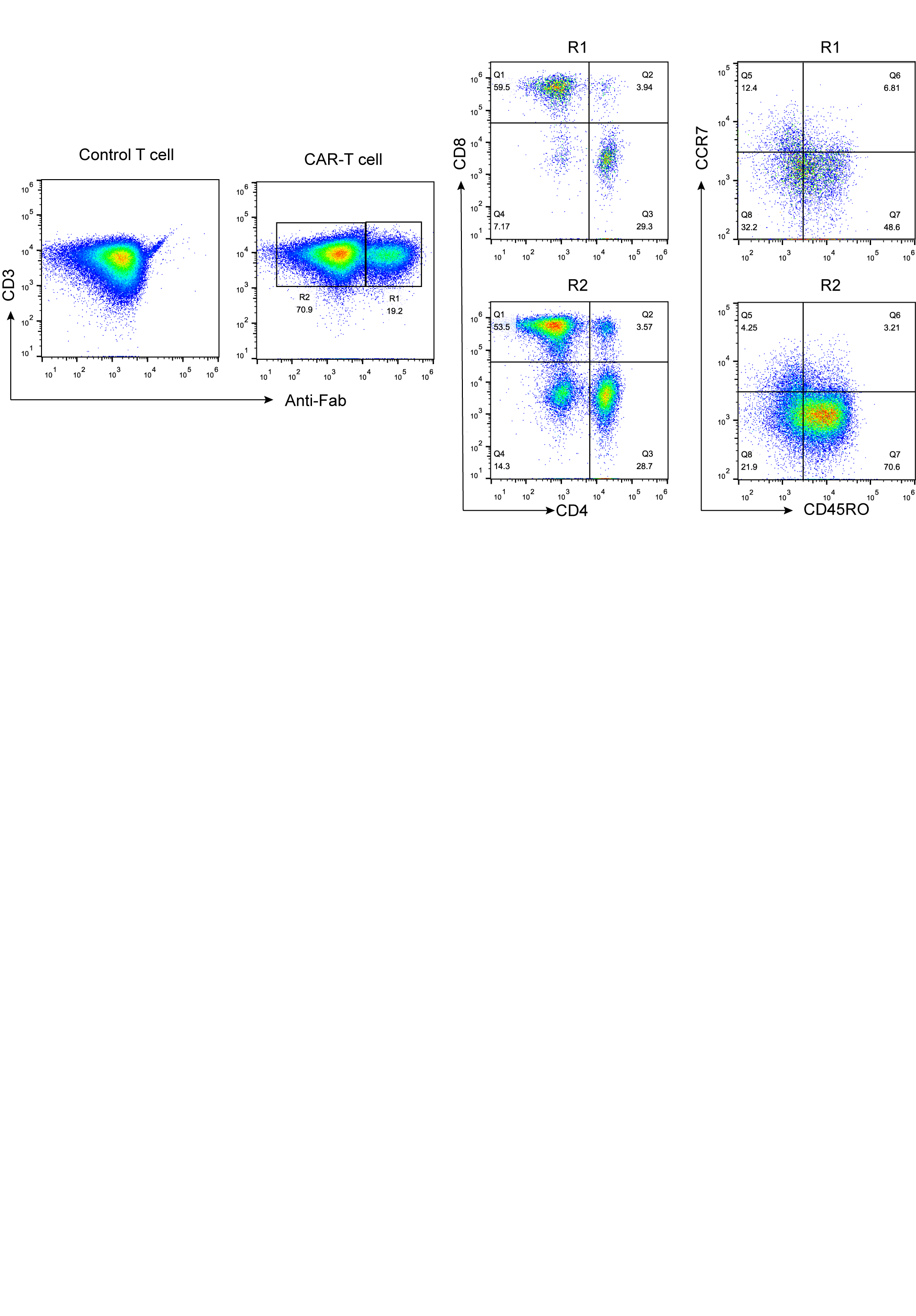


**Figure S3 . The immunophenotype of CAR-T cells**. R1 and R2 showed the expression of CD4, CD8, CD45RO and CCR7 on CAR-T cells and T cells respectively.

**Supplemental References**

1. Wang N, Hu X, Cao W, et al. Efficacy and safety of CAR19/22 T-cell cocktail therapy in patients with refractory/relapsed B-cell malignancies. Blood 2020, 135(1): 17-27.
2. Xu H, Wang N, Cao W, et al. Influence of various medium environment to in vitro human T cell culture. *In Vitro Cell Dev Biol Anim*. 2018.
3. Xu H, Cao W, Huang L, et al. Effects of cryopreservation on chimeric antigen receptor T cell functions. *Cryobiology*. 2018;83:40-47.
4. Biddison WE, Lichtenfels R, Adibzadeh M, et al. Measurement of polyclonal and antigen-specific cytotoxic T cell function. Curr Protoc Immunol. 2001 May;Chapter 7:Unit 7.17.
